# Safety and efficacy of ketorolac in improving the prognosis of acute type A aortic dissection patients: a protocol of a randomized, double-blinded, and placebo-controlled study

**DOI:** 10.1186/s13063-024-08093-x

**Published:** 2024-04-10

**Authors:** Zhikang Lv, Tuo Pan, Haitao Zhang, Yapeng Wang, Yusanjian Matniyaz, Yuxian Tang, Lichong Lu, Dongjin Wang

**Affiliations:** 1 Department of Cardiac Surgery, Nanjing Drum Tower Hospital, Affiliated Hospital of Medical School, Nanjing University, Nanjing, China; 2 Department of Cardiac Surgery, Nanjing Drum Tower Hospital, Chinese Academy of Medical Science & Peking Union Medical College, Nanjing, China; 3 Department of Cardiac Surgery, Nanjing Drum Tower Hospital Clinical College of Nanjing University of Chinese Medicine, Nanjing, China

**Keywords:** Ketorolac, Stanford type A aortic dissection, Prognosis

## Abstract

**Background:**

Acute type A aortic dissection (aTAAD) is a critical and life-threatening condition. Previous research has demonstrated that the use of ketorolac not only reduces the progression, incidence, and severity of aortic aneurysms in animal models, but also decreases postoperative mortality and complications in patients undergoing open abdominal aortic aneurysm replacement. However, there is a lack of studies investigating the efficacy of ketorolac in treating aTAAD in humans. Therefore, we conducted a study to evaluate the safety and efficacy of ketorolac in patients with aTAAD. Our hypothesis was that ketorolac treatment for aTAAD patients would meet safety indicators and effectively improve patient prognosis.

**Methods/design:**

This study is a single-center, randomized, double-blinded, and placebo-controlled study. A total of 120 patients with aTAAD will be recruited and will be randomized into the ketorolac group and placebo group with a ratio of 1:1. Ketorolac tromethamine 60 mg per 2 ml will be intramuscularly injected within 2 h before surgery, followed by intramuscular injections of 30 mg per 1 ml BID. on the first and second postoperative days in the Ketorolac group, while 0.9% saline will be administered at the same dose, dosage form, and time in the placebo group. This study aims to evaluate the safety and efficacy of ketorolac in improving the prognosis of aTAAD. The primary endpoint is the composite endpoint event concerning drug-related adverse events. Secondary endpoints include drug-related adverse events, laboratory examination of blood, diagnostic imaging tests, clinical biomarkers, etc.

**Discussion:**

This study has been approved by the Medical Ethics Committee of Affiliated Nanjing Drum Tower Hospital, Nanjing University Medical College (approval number: 2023–197-02). This study is designed to evaluate the safety and efficacy of ketorolac in patients with aTAAD. All participating patients will sign an informed consent form, and the trial results will be published in international peer-reviewed journals.

**Trial registration:**

The Chinese Clinical Trial Registry (http://www.chictr.org.cn) ChiCTR2300074394. Registered on 4 October 2023.

## Administrative information

Note: the numbers in curly brackets in this protocol refer to SPIRIT checklist item numbers. The order of the items has been modified to group similar items (see http://www.equator-network.org/reporting-guidelines/spirit-2013-statement-defining-standard-protocol-items-for-clinical-trials/).

**Table:** 

Title {1}	Safety and efficacy of ketorolac in improving the prognosis of acute type A aortic dissection patients: a protocol of a randomized, double-blinded, and placebo-controlled study
Trial registration {2a and 2b}.	The Chinese Clinical Trial Registry (http://www.chictr.org.cn) ChiCTR2300074394. Registered on 4 October 2023.
Protocol version {3}	Version 1 of 22–11-2023
Funding {4}	This research is funded by National Natural Science Foundation of China (Project approval number: 82241212) (https://grants.nsfc.gov.cn.)
Author details {5a}	Zhi-Kang Lv: Department of Cardiac Surgery, Nanjing Drum Tower Hospital, Affiliated Hospital of Medical School, Nanjing University, Nanjing, China Tuo Pan: Department of Cardiac Surgery, Nanjing Drum Tower Hospital, Chinese Academy of Medical Sciences& Peking Union Medical College, Graduate School of Peking Union Medical College, Beijing, China Hai-Tao Zhang: Department of Cardiac Surgery, Nanjing Drum Tower Hospital, Chinese Academy of Medical Sciences& Peking Union Medical College, Graduate School of Peking Union Medical College, Beijing, China Ya-Peng Wang: Department of Cardiac Surgery, Nanjing Drum Tower Hospital, Chinese Academy of Medical Sciences& Peking Union Medical College, Graduate School of Peking Union Medical College, Beijing, China Yusanjian Matniyaz: Department of Cardiac Surgery, Nanjing Drum Tower Hospital, Affiliated Hospital of Medical School, Nanjing University, Nanjing, China Yu-Xian Tang: Department of Cardiac Surgery, Nanjing Drum Tower Hospital Clinical College of Nanjing University of Chinese Medicine, Nanjing, China Li-Chong Lu: Department of Cardiac Surgery, Nanjing Drum Tower Hospital, Beijing, China Dong-Jin Wang: Department of Cardiac Surgery, Nanjing Drum Tower Hospital, Affiliated Hospital of Medical School, Nanjing University, Nanjing, China
Name and contact information for the trial sponsor {5b}	Dong-Jin Wang (Principal Investigator) dongjin_wang@126.com
Role of sponsor {5c}	This is an investigator-initiated clinical trial. Therefore, the funders played no role in the design of the study and collection, analysis, and interpretation of data and in writing the manuscript.

## Introduction

### Background and rationale {6a}

Acute type A aortic dissection (aTAAD) is considered the most perilous form of aortic disease [1]. Common causes include hypertension, medial degeneration (cystic medial necrosis), and Marfan syndrome [2]. Currently, rapid surgical reconstruction remains the primary treatment option for aTAAD [3–6]. Based on reported references, the in-hospital mortality rate after surgery stands at 25% [7]. Consequently, there is a pressing need to investigate alternative approaches that can effectively decrease surgical mortality.

Previous studies have shown that activated macrophages in mice secrete inflammatory factors, which can enhance migration and invasion capabilities, accelerate degradation of the aortic media, and contribute to the development of type A aortic dissection (TAAD) [8–10]. Additionally, Rho GTPase, an important regulator of cytoskeleton actin and intracellular signal transduction, has been found to be activated in TAAD [11, 12]. In the literature, it has been observed that ketorolac, a nonsteroidal anti-inflammatory drug and inhibitor of the Rho GTPase signaling pathway, can reduce the progression, incidence, and severity of aortic disease in animal models [13, 14].

Previous clinical research has suggested that ketorolac may reduce postoperative mortality, neurological complications, renal complications, and respiratory failure in patients undergoing open abdominal aortic aneurysm replacement surgery [15]. However, it is important to note that this study was retrospective and focused specifically on abdominal aortic aneurysm. Additionally, the 2022 American Heart Association (AHA) guidelines have reported controversy regarding the use of ketorolac in the analgesic treatment of aTAAD [16]. Therefore, our plan is to conduct a single-center, randomized, double-blinded, and placebo-controlled trial to investigate the safety and efficacy of ketorolac in improving the prognosis of aTAAD patients.

### Objectives {7}

The objective of this study is to assess the safety and efficacy of ketorolac in improving the prognosis of aTAAD.

### Trial design {8}

This study adopts a single-center, parallel-group design, employing randomization, double-blinding, and a placebo-controlled approach. It is a superiority test. Patients diagnosed with aTAAD will be actively recruited and subsequently randomized into either the ketorolac group or the placebo group, maintaining a 1:1 ratio. The study encompasses a follow-up period of 90 days.

## Methods: participants, interventions, and outcomes

### Study setting {9}

This trial is conducted at a single center, specifically at the Department of Cardiac Surgery, Nanjing Drum Tower Hospital. Nanjing Drum Tower Hospital serves as a prominent heart center in Nanjing city, located in Jiangsu province, China. Inclusion criteria, as delineated below, are applied to assess the eligibility of patients for recruitment into the study.

### Eligibility criteria {10}

The inclusion criteria are as follows:

1. Patients diagnosed with aTAAD require emergency operation;
2. Patients aged between 18 and 65; and
3. Signing an informed consent.

The exclusion criteria are as follows:

1. Long-term fasting or inability to self eat;
2. History of malignant tumors;
3. Weight < 50 kg;
4. Traumatic aortic dissection;
5. Patients with Marfan syndrome;
6. Unstable vital signs require mechanical assistance or rescue before surgery (IABP, ECMO, LVAD, etc.);
7. Patients required endotracheal intubation before surgery;
8. After admission, patients with consciousness disorder, central nervous system dysfunction, or evidence of cerebral hypoperfusion;
9. Preoperative hematemesis, black or bloody stools, or bowel dilation;
10. Limb ischemia before surgery;
11. Malperfusion syndrome before surgery;
12. Patients who need percutaneous interventions to relieve malperfusion;
13. History of digestive ulcer or chronic gastroenteritis;
14. Dialysis before admission or a history of renal insufficiency;
15. History of liver disease;
16. Allergies to aspirin and non-steroidal anti-inflammatory drugs like ketorolac tromethamine;
17. Chronic inflammatory diseases, autoimmune diseases, or other situations require long-term use of hormones or non-steroidal drugs;
18. No cerebral perfusion during deep hypothermic circulatory arrest;
19. History of Grade 4 surgery or acute myocardial infarction within 90 days;
20. History of cardiac surgery or operations on great vessels;
21. Pregnant and lactating women;
22. Refusing to participate in this clinical trial or sign an informed consent form;
23. Other situations where the subject deems it unsuitable to participate in this project.

### Who will take informed consent? {26a}

Patients diagnosed with aTAAD and planning to undergo surgical treatment will undergo eligibility screening to determine their suitability for participation in this trial. Once the surgeon has assessed the patient as eligible, their family will be invited to meet with the research physician. During this meeting, their family will have the opportunity to ask any questions they may have and sign the informed consent form.

### Additional consent provisions for collection and use of participant data and biological specimens {26b}

Consent will be sought for the retrospective review of participants’ medical records and the procurement of blood samples to evaluate inflammation, document adverse events, conduct comprehensive blood laboratory examinations, and analyze clinical biomarkers.

## Interventions

### Explanation for the choice of comparators {6b}

Patients diagnosed with acute type A aortic dissection (aTAAD) will be randomly allocated to either the ketorolac group or the placebo group, maintaining a 1:1 ratio. The intervention entails the administration of ketorolac tromethamine or 0.9% saline to the respective subjects. Ketorolac tromethamine, serving as the interventional treatment in the trial, will be administered 2 h prior to surgery (60 mg per 2 ml) and will continue for the initial 2 days post-operation (30 mg per 1 ml BID). The use of 0.9% saline functions as an inert placebo in the trial.

### Intervention description {11a}

The surgeries will be performed by Professor Dongjin Wang. The study will commence on the day the patients sign an informed consent form and receive their randomized numbers. The ketorolac group, consisting of patients with aTAAD, will be treated with ketorolac. They will receive a preoperative intramuscular injection of 60 mg per 2 ml ketorolac and a postoperative injection of 30 mg per 1 ml ketorolac BID for two consecutive days. The placebo group, also consisting of patients with aTAAD, will receive placebo treatment. They will receive a preoperative intramuscular injection of 2 ml 0.9% saline placebo and a postoperative injection of 1 ml 0.9% saline placebo BID for two consecutive days. During the administration of ketorolac, patients’ blood pressure and renal function will be routinely monitored, and targeted treatment methods such as sedation, hypotension, diuresis, and dialysis will be applied as necessary. Additionally, all included patients will receive the same medical and surgical treatments in accordance with the diagnosis and treatment guidelines for aTAAD. Aortic dissection tissue samples will be collected for laboratory study during the surgery. Both the ketorolac group and the placebo group patients will undergo follow-up examinations before surgery and on the 1st, 3rd, 5th, 7th, 30th, and 90th day after surgery. These examinations will include assessments of adverse drug events, cardiac ultrasound, chest X-ray, laboratory tests (blood routine examination, blood biochemistry, erythrocyte sedimentation rate, coagulation function, CK-MB, cardiac troponin I (cTnI), PCT, IL-6, CRP, myohemoglobin (MYO), type B natriuretic peptide (BNP), D-dimer, etc.) and clinical biomarkers (matrix metalloproteinase-9 (MMP-9), MMP-2, MMP-3, MMP-13, type III procollagen, etc.). CTA examination of the cervical, thoracic, and abdominal aorta artery will be performed before surgery and on the 30th and 90th days after surgery.

### Criteria for discontinuing or modifying allocated interventions {11b}

Subjects can withdraw from the study at any time for any reason. Considering the safety of the subjects. When the subject experiences situations where it is not appropriate to continue the study, including worsening of the condition, serious adverse events, poor compliance, experiencing intolerable accidents such as bankruptcy or family misfortune, and unwillingness to sign an informed consent form. Patients can be terminated to continue this study.

### Strategies to improve adherence to interventions {11c}

The administration of drugs and the collection of blood and tissue samples will be closely monitored by our specialized staff at our center. They will maintain close communication with the treating nurse and surgeons and monitor the progress of the trial.

### Relevant concomitant care permitted or prohibited during the trial {11d}

All included patients will receive the same medical and surgical treatments in accordance with the diagnosis and treatment guidelines for aTAAD.

### Provisions for post-trial care {30}

The sponsor has insurance that covers damage to research subjects resulting from injury or death caused by ketorolac. This insurance applies to any damage that becomes apparent during the trial and the subsequent 90-day follow-up period.

### Outcomes {12}

#### Primary endpoint

Efficacy: death, organ malperfusion syndrome, permanent dialysis, tracheotomy, neurological impairment, postoperative mechanical circulatory support, and unplanned cardiac reoperation.

Safety: gastrointestinal ulceration, gastrointestinal bleeding, gastrointestinal perforation, postoperative hemorrhage (postoperative hemorrhage exceeding 1000 ml within 24 h), renal failure, liver failure, drug allergies, and other severe adverse events following drug administration.

#### Secondary endpoints

1. Erythrocyte sedimentation rate (ESR), C-reactive protein, procalcitonin, SII [17], coagulation function (prothrombin time, activated partial prothrombin time, bleeding time), liver function (alanine transaminase, glutamic transaminase, lactate dehydrogenase, total bilirubin, direct bilirubin), renal function (creatinine, uric acid, eGFR), high-sensitivity troponin T, serum B-type natriuretic peptide, myocardial enzyme (creatine kinase, creatine kinase MB isoenzyme), and other laboratory examination of blood;
2. Chest X-ray (for pulmonary inflammation and effusion, etc.), CTA scan of the cervical, thoracic, and abdominal aorta artery (for aortic diameter and extent of dissection, etc.), cardiac ultrasound (for cardiac ejection fraction and left ventricular diastolic dysfunction, etc.), and other imaging examinations.
3. The patient’s cardiopulmonary bypass time during surgery, total surgical time, aortic cross-clamping time, and postoperative hospital stay;
4. Postoperative drainage color, drainage volume, postoperative patient blood transfusion, and transfusion volume;
5. The incidence of postoperative sternal infection, secondary debridement, secondary tracheal intubation, pneumonia, and delirium; and
6. Enzyme-linked immunosorbent assay (ELISA) will be performed to detect and measure IL-1β IL-2, IL-4, IL-6, IL-8, IL-10, IL-12, IL-13, TNF-α, INF-γ, granulocyte–macrophage colony-stimulating factor (GM-CSF) and calcitonin;

## Safety assessment

Ketorolac has been used in clinical practice for many years and is generally well tolerated. According to the medication instructions, the primary adverse reactions associated with ketorolac are gastrointestinal reactions, gastric bleeding, and potential liver and kidney damage. Guidelines from the AHA suggest that ketorolac may also contribute to hypertension and kidney damage [16]. And some cardiovascular adverse events related to ketorolac should be included. Therefore, the safety assessment variables include death, organ malperfusion syndrome, hypertension (blood pressure > 140/90 mmHg), stage 2 AKI diagnosed by KIDGO criteria [18], tracheotomy, neurological impairment prior to discharge, postoperative mechanical circulation support, and postoperative cardiac arrest.

### Participant timeline {13}

Table 1 shows the participant timeline.

**Table 1:** Participant timeline

	Study period								
	Enrolment	Allocation	Post-allocation						Close-out
Timepoint	Before surgery	Before surgery	During surgery	Postoperative day 1	Postoperative day 3	Postoperative day 5	Postoperative day 7	Postoperative day 30	Postoperative day 90
Enrolment									
Informed consent	** × **								
Eligibility screen	** × **								
Allocation		** × **							
Randomization		** × **							
Interventions									
Tissue samples collection			** × **						
Ketorolac tromethamine		** × **		** × **	** × **				
0.9% saline		** × **		** × **	** × **				
Assessments:									
Vital signs	** × **		** × **	** × **	** × **	** × **	** × **	** × **	** × **
Hematology examination	** × **		** × **	** × **	** × **	** × **	** × **	** × **	** × **
CTA scan of cervical thoracic and abdominal aorta artery	** × **							** × **	** × **
Chest X-ray				** × **	** × **	** × **	** × **	** × **	** × **
Echocardiography	** × **				** × **			** × **	** × **
Adverse events	** × **		** × **	** × **	** × **	** × **	** × **	** × **	** × **
Complications	** × **		** × **	** × **	** × **	** × **	** × **	** × **	** × **

### Sample size {14}

The required sample size was calculated using the PASS (15.0.5) software. The primary endpoint of efficacy was used to calculate the sample size. In the literature, the total death rate of hospitalization after surgery was 25% [7], the incidence of organ malperfusion syndrome was 15–33% [19, 20], permanent dialysis was 2.6% [21], tracheotomy was 8% [22], neurological impairment was 6.9% [22], postoperative mechanical circulatory support was 20% [23], unplanned cardiac reoperation was 4.2% [24], and postoperative cardiac arrest was 0.7–5.2% [25]. Therefore, we assumed that the incidence of composite endpoint events in the control group is 70%, and the incidence of composite endpoint events in the ketorolac group is reduced to 40%. Using PASS (15.0.5) software, when the sample size of the ketorolac group and the control group is 53 cases, this difference can be detected with over 90% confidence. Considering that the loss of follow-up rate is higher than 10%, the final total sample size of this experiment is 120 cases, with 60 cases in the experimental group and 60 cases in the control group.

### Recruitment {15}

Commencing from August 2023, all patients with type A aortic dissection (aTAAD) undergoing treatment at our institution will be informed and invited to participate in this clinical trial. The study aims to enroll a total of 120 eligible patients from the cardiothoracic surgery department of Nanjing Drum Tower Hospital within the timeframe spanning August 2023 to December 2026. Our center, renowned for its expertise, conducts more than 250 surgical procedures for aTAAD annually. Anticipating a robust enrollment process, we project the successful recruitment of the targeted 120 patients within the designated timeframe.

## Assignment of interventions: allocation

### Sequence generation {16a}

This study employs a block-randomized treatment allocation strategy to minimize selective bias in treatment assignment. The trial utilizes a block randomization approach, facilitated by SAS 9.4 statistical software, with participants allocated in a 1:1 ratio to the ketorolac and placebo groups. Sequentially numbered random codes are generated based on the “Central Code Random Number Table.” The random number table is provided by qualified professionals. As recruitment is finishing, participants can only obtain a random code, without knowledge of the corresponding medication.

### Concealment mechanism {16b}

This trial employs a double-blind design to ensure the implementation of blinding throughout the experimental process. We conduct medication blinding based on randomized codes. Ketorolac and placebo drugs will be enclosed in identical envelopes. Participants, drug dispensing center staff, and trial personnel will be unable to discern the type of medication based on envelope appearance. Blinding will be accomplished by encoding the experimental and control drugs according to random information and placing them into indistinguishable envelopes. Each coded drug will have an accompanying emergency letter for unblinding in urgent situations. Monitors and researchers must remain blinded throughout, and records of the blinding process will be meticulously maintained.

After obtaining informed consent forms, clinical physicians, responsible for recruiting participants based on inclusion criteria, will sequentially assign random codes. A drug administrator, independent from the study intervention and evaluation, will dispense the drug based on the random code. The drug, enveloped in a standardized cover, will then be administered to the patient. The names and random codes of participants will be recorded and stored by clinical physicians, while the drug administrator will handle drug distribution and retrieval. Records of both dispensing and retrieval will be documented. Until the trial is unblinded, all involved personnel will remain unaware of the grouping of participants.

### Implementation {16c}

Essentially, qualified professionals generate random codes, and a subgroup not involved in medication distribution independently packages and codes the drugs according to these random codes. This ensures a one-to-one correspondence between the random codes and the medications. Upon patient enrollment, the clinical doctor assesses eligibility, and patients are assigned sequential random codes. The medication administrator then dispenses the drugs based on these random codes.

## Assignment of interventions: blinding

### Who will be blinded {17a}

This study adheres to a double-blinded design, ensuring that both the study staff (excluding those engaged in drug preparation) and the patients remain unaware of the treatment assignments.

### Procedure for unblinding if needed {17b}

Emergency unblinding can be performed at any time if patients experience severe adverse events or unexpected worsening of their clinical status, as deemed necessary by the principal investigator or the sponsor. Participants are provided with “In case of emergency” cards, which should be carried at all times during the study and include an emergency phone number. The researcher must record the date, location, reason, person responsible for unblinding, main investigator, and relevant personnel in charge of the drug clinical trial institution. These details should be documented in the case report form (CRF). Subjects who undergo emergency unblinding will be considered as withdrawal cases.

## Data collection and management

### Plans for assessment and collection of outcomes {18a}

Clinical data, including patient admission information, demographic data, laboratory blood examinations, and imaging examinations, will be collected from the electronic medical record (EMR). The CRFs will be initially filled out on paper and then entered into Microsoft Excel by the associated researchers. The follow-up period will last for 90 days, during which the researchers will record all medical data and laboratory results from any hospital visits. At the end of the follow-up period, the patients will be interviewed via telephone.

### Plans to promote participant retention and complete follow-up {18b}

Telephone follow-up will be systematically conducted to prompt patients to attend follow-up appointments at 30 and 90 days post-surgery. Additionally, the trial will offer patients financial assistance, encompassing travel expenses and accommodations.

### Data management {19}

Patient data will be initially sourced from the Electronic Medical Record (EMR) and subsequently transcribed onto the Case Report Form (CRF). The patient data will be securely stored in Excel format on a computer. The CRF will meticulously capture specific details of adverse events (AEs) experienced by the patients.

To ensure the quality of trial data, we employ data range checks. Prior to the commencement of the trial, expected ranges for each measurement variable are established, grounded in prior research, clinical guidelines, or professional consensus. These ranges are designed to be both reasonable and aligned with clinical practice. Pre-data entry, range checks are executed to eliminate potential human errors. Post data entry, the utilization of graphical tools and visualization methods such as histograms and box plots aids in identifying outliers or issues with data distribution. We also have dedicated data monitors who randomly inspect the CRFs completed by study personnel. Any identified outliers will be promptly documented and reported.

### Confidentiality {27}

The original Case Report Forms (CRFs) and Informed Consent Forms (ICFs) will be securely stored in a locked filing cabinet within the Department of Cardio-Thoracic Surgery at Nanjing Drum Tower Hospital, The Affiliated Hospital of Nanjing University Medical School. Only data monitors possess the authorization to access and review the hard-copy documents within the filing cabinet. Patient identification details will not be disclosed in any publications. Following the publication of the article, trial data will be shared with other researchers through secure electronic means, such as email, to facilitate international prospective meta-analyses.

### Plans for collection, laboratory evaluation, and storage of biological specimens for genetic or molecular analysis in this trial/future use {33}

All patients will provide aortic dissection tissue samples during surgery, which will be sent to the laboratory for hematoxylin–eosin staining, western blot, and immunofluorescence. Additionally, blood samples will be collected before surgery and at 1, 3, 5, and 7 days after surgery. These blood samples will also be sent to the laboratory for ELISA. The tissue specimens will be stored at room temperature after being embedded in paraffin or frozen in liquid nitrogen and stored in a refrigerator at − 80 ℃. Similarly, the blood samples will be centrifuged and stored in a refrigerator at − 80 ℃.

## Statistical methods

### Statistical methods for primary and secondary outcomes {20a}

Statistical analysis will be performed using SPSS software (version 26.0) and R (× 64 3.5.0). Continuous variables will be presented as mean ± standard deviation. The Kolmogorov–Smirnov test will assess normal distribution. If variables exhibit normal distribution, an independent sample *t*-test will be employed; otherwise, the Mann–Whitney *U* test will be utilized. Categorical variables’ frequency will be expressed as percentages and analyzed using the chi-square test or Fisher’s exact test. It should be noted that we are not conducting an Intention to Treat analysis due to the exclusion of patients with more than 10% missing data. Double-tailed *p*-values and 95% confidence intervals will be reported, with statistical significance defined as *p* < 0.05.

### Interim analyses {21b}

Interim analysis will be performed. When we complete the enrollment of 60 patients, 30 of whom are in the ketorolac group and 30 in the placebo group, we will perform an interim analysis.

### Methods for additional analyses (e.g., subgroup analyses) {20b}

There are no subgroup analyses planned.

### Methods in analysis to handle protocol non-adherence and any statistical methods to handle missing data {20c}

We allow patients to have 10% missing data, which will be deleted as blank. When the patient’s data is missing by more than 10%, the patient will be deleted.

### Plans to give access to the full protocol, participant-level data, and statistical code {31c}

The datasets used and/or analyzed during the current study can be made available by the corresponding author upon reasonable request.

## Oversight and monitoring

### Composition of the coordinating center and trial steering committee {5d}

This study is a single-center study conducted at Nanjing Drum Tower Hospital. The trial involves various roles:

- Principle investigator: oversees the trial and has medical responsibility for the patients.
- Data manager: organizes data capture and ensures data quality.
- Study coordinator: handles trial registration, coordinates study visits, and prepares annual safety reports.
- Study physician: obtains informed consent, and ensures follow-up according to the protocol.
- A research assistant: randomizes the patients and administers the drug.
- Statistician: creates random scales and provides consultation on statistical methods.
- The study team holds weekly meetings. There is no trial steering committee or stakeholder and public involvement group.

### Composition of the data monitoring committee, its role and reporting structure {21a}

The Data and Safety Monitoring Board (DSMB) is composed of a chair, who is a cardiologist, along with a cardiac surgeon, an independent statistician, a cardiac anesthesiologist with expertise in medical ethics and law, and a clinical pharmacologist. The DSMB will formulate tailored data security monitoring plans, taking into consideration the magnitude of associated risks. Given the high-risk nature of this study, an autonomous Data Security Monitoring Committee has been established to systematically oversee the accumulated data on security and effectiveness. The committee’s mandate is to provide well-informed recommendations regarding the continuation of the research.

### Adverse event reporting and harms {22}

Any adverse events (AEs) that occur during the study period will be carefully documented, evaluated, and treated. AEs grading will be based on Common Terminology Criteria for Adverse Events V.5.0.In the case of serious AEs that pose a risk to the patient’s life, an immediate report will be submitted to the Medical Ethics Committee of Affiliated Nanjing Drum Tower Hospital, Nanjing University Medical College. Additionally, the incidence of AEs will be calculated.

### Frequency and plans for auditing trial conduct {23}

An independent study monitor will be appointed to conduct study-specific auditing. The monitor will visit one time for every 40 patients and verify the presence and completeness of the investigation file. Additionally, the monitor will review the following data for 25% of randomly selected patients: informed consents, inclusion and exclusion criteria, source data, and documentation of any missing or reported AEs.

### Plans for communicating important protocol amendments to relevant parties (e.g., trial participants, ethical committees) {25}

A “substantial amendment” is defined as an amendment that is likely to have a significant impact on various aspects of a trial. These aspects include the safety and well-being of the trial participants, the scientific value of the trial, the overall conduct and management of the trial, as well as the quality and safety of any intervention used in the trial. Whenever a substantial amendment occurs, it is mandatory to notify both the Medical Ethics Committee of Affiliated Nanjing Drum Tower Hospital, Nanjing University Medical College, and the competent authority. On the other hand, non-substantial amendments are recorded and filed for documentation purposes. If any amendments directly affect or involve the participants, they are duly informed about the changes. In such cases, if necessary, additional consent will be requested and properly documented. Furthermore, it is essential to update the online trial registries to reflect the amendments made.

### Dissemination plans {31a}

The findings of this research will be comprehensively disseminated through international peer-reviewed journals. The reporting will encompass both positive and negative outcomes. Additionally, a lay summary will be crafted, designed to be shared with all participating families, providing accessible information about the research outcomes.

## Discussion

This study presents the first randomized, double-blinded, and placebo-controlled trial conducted to evaluate the safety and efficacy of ketorolac in the treatment of aTAAD. The findings of this trial will serve as a valuable reference for the use of ketorolac in the treatment of aTAAD. If the hypothesis of this trial proves to be accurate, ketorolac treatment for aTAAD can be considered safe and potentially beneficial in reducing inflammatory biomarkers, such as IL-6, CRP, and PCT.

The guidelines state that ketorolac can increase hypertension and cause renal dysfunction, so patients with acute aortic syndrome should use it with caution [16]. However, the guidelines only mention aortic disease and do not specifically address the potential harm of ketorolac in aTAAD. There is also no clear literature support provided. Previous clinical studies have shown that ketorolac is safe for perioperative treatment of surgery [26], and it has also been found to be beneficial in the treatment of aTAAD [15]. Our preliminary basic experiments have confirmed the beneficial effects of ketorolac on animal aortic aneurysms, which align with the conclusions of published basic research papers [12, 27, 28]. From a clinical application perspective, patients in the ICU undergo strict monitoring of their blood pressure, urine output, etc. Active treatment methods can effectively control the hypertension and kidney damage that are concerning in the guidelines. Therefore, the use of ketorolac in the treatment of aTAAD remains a controversial issue.

We intend to reevaluate the safety and efficacy of ketorolac in the treatment of aTAAD. Our approach involves two main components: Firstly, we will conduct a randomized controlled trial. Secondly, we will collect aortic tissue samples from patients during surgery and perform a series of laboratory experiments to investigate the underlying mechanisms and pathways. By integrating both clinical trials and basic experiments, our study aims to offer scholars a solid reference framework and novel insights.

## Trial status

The trial started patient recruitment in August 2023. The current protocol is version 2 of 20–3-2024. Currently (20th of March 2024), we have included fifty-five patients. The recruitment process will persist for a duration of 2 years, concluding in December 2026.

## Data Availability

Upon reasonable request, the corresponding author will provide access to the datasets utilized and/or examined during the present investigation.

## Abbreviations

AEs: Adverse events
AHA: American Heart Association
aTAAD: Acute type A aortic dissection
BNP: Type B natriuretic peptide
CRF: Case report form
cTnI: Cardiac troponin I
DSMB: Data and safety monitoring board
ELISA: Enzyme-linked immunosorbent assay
EMR: Electronic medical record
ESR: Erythrocyte sedimentation rate
GM-CSF: Granulocyte-macrophage colony-stimulating factor
ICFs: Informed consent forms
MMP-9: Matrix metalloproteinase-9
MYO: Myohemoglobin
PAR1: Protease-activated receptor-1
qPCR: Quantitative real-time polymerase chain reaction
TAAD: Type A aortic dissection
